# Determinants of low family planning use and high unmet need in Butajira District, South Central Ethiopia

**DOI:** 10.1186/1742-4755-8-37

**Published:** 2011-12-08

**Authors:** Wubegzier Mekonnen, Alemayehu Worku

**Affiliations:** 1 School of Public Health, College of Health Sciences, Addis Ababa University, Addis Ababa, Ethiopia

**Keywords:** Family planning use, unmet need, Butajira, rural, Ethiopia

## Abstract

**Background:**

The rapid population growth does not match with available resource in Ethiopia. Though household level family planning delivery has been put in place, the impact of such programs in densely populated rural areas was not studied. The study aims at measuring contraception and unmet need and identifying its determinants among married women.

**Methods:**

A total of 5746 married women are interviewed from October to December 2009 in the Butajira Demographic Surveillance Area. Contraceptive prevalence rate and unmet need with their 95% confidence interval is measured among married women in the Butajira district. The association of background characteristics and family planning use is ascertained using crude and adjusted Odds ratio in logistic regression model.

**Results:**

Current contraceptive prevalence rate among married women is 25.4% (95% CI: 24.2, 26.5). Unmet need of contraception is 52.4% of which 74.8% was attributed to spacing and the rest for limiting. Reasons for the high unmet need include commodities' insecurity, religion, and complaints related to providers, methods, diet and work load. Contraception is 2.3 (95% CI: 1.7, 3.2) times higher in urbanites compared to rural highlanders. Married women who attained primary and secondary plus level of education have about 1.3 (95% CI: 1.1, 1.6) and 2 (95% CI: 1.4, 2.9) times more risk to contraception; those with no child death are 1.3 (95% CI: 1.1, 1.5) times more likely to use contraceptives compared to counterparts. Besides, the odds of contraception is 1.3 (95% CI: 1.1, 1.6) and 1.5 (1.1, 2.0) times more likely among women whose partners completed primary and secondary plus level of education. Women discussing about contraception with partners were 2.2 (95% CI: 1.8, 2.7) times more likely to use family planning. Nevertheless, contraception was about 2.6 (95% CI: 2.1, 3.2) more likely among married women whose partners supported the use of family planning.

**Conclusions:**

The local government should focus on increasing educational level. It must also ensure family planning methods security, increase competence of providers, and create awareness on various methods and their side effects to empower women to make an appropriate choice. Emphasis should be given to rural communities.

## Background

The rapid population growth does not match with available resource in Ethiopia [1] where the economy has been agrarian based on household subsistence farming [2]. Agricultural households in the country whose livelihoods depend on petty mixed farming require involvement of many household members including children who work on laborious farms free of pay. In this situation, large family size has been an accepted norm in Ethiopia to divide demanding farm workload among family members. The country also has a young population due to mainly high fertility in rural areas in the country [3-5].

Family planning is a viable solution to control such fast growing populations. In addition to spacing and limiting the number of children it improves maternal and child health, empowers women and enhances economic development [6]. However, married women of reproductive age group have unmet need for contraception, inability to use family planning methods to prevent pregnancy despite the interest of practicing it.

Contraception and unmet need levels varied across countries of the world, the lowest contraception and highest unmet need, being in sub-Saharan Africa mainly because of low level of knowledge and existence of variety of barriers [7,8]. Knowledge of any modern method among married women in Ethiopia increased from 85% in 2000 to 87% in 2005 [9,10] though contraception increased to only 15% in 2005 from its lowest level of 4% in 1990 [1,9,10]. The national contraceptive prevalence rate has also been more than doubled to about 29% in 2011[11]. Regional disparities in knowledge, access and use of family planning methods have also been observed in the country. Contraception among married women in the study region, where Butajira district is located, was consistently lower than the national level across the three rounds of demographic and health survey years [9-11]. On the other hand, unmet need for family planning was estimated at 35.8%, 33.8 and 25.3% in 2000, 2005 and 2011 respectively in Ethiopia. Unmet need varies across regional states in the country. Its value in the study region was similar to the national level except for 2005 which was by 3.6% higher than the country's unmet need. About 60.9%, 59.4% and 64.4% of the national unmet need for contraception in 2000, 2005 and 2011 respectively are attributed to child spacing. On the same token, the share of unmet need for spacing from the total unmet need was higher for SNNPR compared to the national except for 2011. Disaggregation of unmet need by population characteristics revealed significantly higher level among young women, living in rural areas, resides in Oromia and the study regions, illiterates and living in households with lowest wealth quintiles in 2005 [10].

Social and economic development of countries and communities dictated the level of contraception and reasons for unmet need [12]. Various population background characteristics related to health, population and economic policy environments, household structure and individual characteristics were associated to contraception [13]. Absence of all and/or certain family planning methods in the area, costs related to family planning methods provision, women's autonomy and their socio-economic status, medical and legal restrictions to use methods and provider bias and misinterpretation were shown as barriers of contraception [14]. Moreover, educational status of women and their partners, socio-economic level, type of residence area, access to media, knowledge about family planning methods, support to family planning methods by women and/or their partners and senior members of their family, age, migration status, wealth equity, parity, experience of abortion and child death, religious beliefs, ethnic affiliations were some of the individual background characteristics that were associated to the use of family planning methods [13,15-18]. On the other hand, reproductive health interventions changes the level of current use of contraceptives among women of reproductive age group and their partners [19]. The distal socio-demographic, economic, cultural and environmental individual factors have been indirectly influencing family planning use through proximate factors such as discussion between partners, knowledge of methods and their sheer availability and mix, distance of service delivery points and skills of providers to persuade their clients on correct and sustainable use of family planning methods [20,21].

Although family planning use among women of reproductive age has been increasing from its virtually non-existence level, it is still low in Ethiopia. Contraception has been consistently lower while unmet need is higher in the region where Butajira district is located compared to the national level. Besides, the nationwide health extension program (HEP) launched in 2003, showed a low success rate in 2008 in the study region[22,23]. Family planning provision to rural communities in the country is one of the 16 modules in HEP. Village based female health extension workers are overburdened with many activities which may deter the program's success.

Butajira district is predominantly rural, suffered from population pressure, inhabited by Muslims; and polygamy and widow inheritance is common [24]. Polygamy was attributed as barrier of contraception among predominantly Muslim communities in Nigeria [25] by raising women's desire to have many children through competition between co-wives to skew household resources towards them. As there is no study on the issue under caption, this study aimed at measuring levels and identifying determinants of contraception and unmet need for family planning use in the context of household level reproductive health delivery in a rural district of Ethiopia.

## Methodology

The study was conducted in the Butajira district. All usual resident women of reproductive age group (15-49 years) within the Demographic Surveillance Area (DSA) of Butajira Demographic Surveillance System (DSS) were interviewed during October to December 2009. Clinical nurses were used as data collectors supervised by experienced staff with health and social science background. A structured Demographic and Health Survey type questionnaire was used to interview women. After they were briefed about the objectives of the study, oral consent were obtained from each study participant. Ethical clearance was also secured from the Institutional Review Board of College of Health Sciences; Addis Ababa University after all ethical issues were considered. A total of 5746 married women were included in this study. Methodological details of the comprehensive study are documented elsewhere [26].

Data were entered and cleaned in EPI INFO software with a CHECK program and analyzed using STATA version 11. Married women are disaggregated by various background characteristics to have an insight of their characteristics. All background characteristics of women used in this study were categorical though the dependant variable, family planning use, was binary in nature. Current contraceptive prevalence rate and unmet need of contraception with 95% confidence interval were computed. Unmet need for contraception was decomposed into unmet need for limiting and spacing. Confidence interval was computed for unmet need for spacing since it had a major share of the total unmet need of contraception. Bivariate and multivariate Logistic regression with odds ratio along with the 95% confidence interval were used to ascertain the association between covariates and family planning use. Only covariates that were statistically significant at the bivariate level were included in the multivariate binary Logistic regression to control for confounding. Though many variables were considered in the analysis, only covariates significantly associated with contraception were reported.

## Results

The 5746 married women varied in background characteristics as shown in Table 1. Married women were fairly distributed in ecological residential areas. About 72% of women never been enrolled into a formal education. Study participants were asked whether their households encountered any food shortage in the past year. They reported that food shortage was common in a third of households while 43% of married women had experience of child death. About 51% of sexual partners completed primary level of education. Nearly 38% of married women mentioned a birth place other than the Demographic Surveillance area. Three-fourth of the women belonged to a household whose main livelihood was farming. Majority were in the age bracket of 20-34 years with the mean age of 30.6 (± 7.8 SD) years (result not shown).

**Table 1:** The frequency distribution of married women by their background characteristics in Butajira, 2009 (N = 5746)

Variable		Number	Frequency
Ecology	Urban	1462	25.5
	Lowland	2128	37.0
	Highland	2156	37.5

Educational status	Never	4153	72.3
	Primary	1310	22.8
	Secondary plus	283	4.9

Household food shortage	Yes	1951	34.0
	No	3794	66.0

Had dead child	No	3084	57.0
	Yes	2329	43.0

Partner's education	Never	2128	38.6
	Primary	2952	51.4
	Secondary plus	576	10.0

Migration status	In-migrant	2165	37.7
	Non-migrant	3580	62.3

Household livelihood	Farming	4296	74.8
	Trade/service	805	14.0
	Civil service	272	4.7
	Other	373	6.5

Discussion	Yes	3820	66.8
	No	1895	33.2

Partner's support	Yes	3899	67.9
	No	1844	32.1

Critical examination of married women by their reproductive health characteristics showed that about two-third of them reported discussion about family planning among couples. Furthermore, about 87% of them (result not shown) and only 68% of their partners supported the use of family planning in Butajira district.

About 99% of women in Butajira district knew at least one method of contraception though some family planning methods were better known than others among resident women of a densely populated Butajira district in Ethiopia as shown in Table 2. Dipo-Provera and Pills were known by more than 97% of married women each followed by male condom and Norplants by about 82% and three-quarter of study participants, respectively. The least known modern contraceptive method was foam/jelly. Emergency contraceptive was also known by about 10% of married women in the study area. Meanwhile, traditional methods such as calendar method, Lactational Amenorrhea Method (LAM) and withdrawal were mentioned by 21.3%, 31% and 20.2% respectively of married women in the district.

**Table 2:** Distribution of knowledge, ever and current use of Family Planning Methods by their type among married women in Butajira district, 2009 (N = 5746)

FP Method type	Percentage of FP Knowledge	Percentage of Ever users	Number of Ever users	Percentage of Current use (n = 1586)
Tubal ligation	19.0	2.9	1089	1.9

Vasectomy	8.2	1.1	473	0.1

Pills	97.5	24.7	5598	10.4

Loop	13.1	3.3	754	0.7

Depo-Provera	97.8	42.2	5619	74.2

Norplant	74.4	9.0	4272	6.1

Condom	81.9	1.7	4707	0.6

Foam/Jelly	4.1	0.9	235	0.0

Calendar Method	21.3	16.5	1222	5.0

LAM	31.0	14.7	1778	1.4

Female Condom	26.6	0.4	1529	0.1

Withdrawal	20.2	12.3	1158	1.1

Emergency contraceptive	10.2	2.0	587	0.1

Total	99.1	51.3	5746	25.4 (24.2, 26.5)

On the other hand, more than half of married women have ever used one or another type of contraceptive. The most favorite modern contraceptive ever practiced among married women were Depo-Provera (42.2%) followed by pills (24.7%). The least ever used modern method of contraceptive was female condoms (0.4%). Emergency contraceptive has ever been used by 2% of married women in the study district. Nevertheless, calendar method, LAM and withdrawal had ever practiced by 16.5%, 14.7% and 12.3% of married women.

Nonetheless, about 25.4% (24.2, 26.5) of married women were currently practicing contraceptives. Still three-fourth of women currently using family planning method prefer Depo-Provera followed by about 10% and 6.1% of women who choose pills and Norplant, respectively. There was no woman among study participants who used foam/jelly for any parity specific control. Among traditional methods, calendar method was currently used by about 5% of women.

Disaggregation of knowledge by type of residential area showed that every woman in urban areas knew about contraceptives while more than 97% of them knew at least one method in lowland or highland Butajira as shown in Table 3. The level of ever and current use of family planning methods among married women by residential ecology was dissimilar. Ever use of family planning methods among married women was more than 77% in urban areas followed by about 45% and 40% of lowlanders and highlanders, respectively.

**Table 3:** The percentage distribution of knowledge, ever and current contraception and unmet need by residence type in Butajira district, 2009 (N = 5746)

Residence Type	**No**.	% of married women Knowing FPM	% of ever use of FPM	% of current use of FPM	% of unmet need for limiting	% of unmet need for spacing (CI)	% of total unmet need (CI)
Urban	1462	100.0	77.4	46.9 (44.3, 49.4)	12.5	25.0 (22.8, 27.3)	37.5 (35.0, 40.0)

Rural low	2128	98.0	44.8	19.5 (17.9, 21.3)	12.7	44.3 (42.2,46.4)	57.0 (54.9, 59.2)

Rural high	2156	99.6	40.1	16.5 (15.0, 18.1)	14.3	43.7 (41.6, 45.8)	58.0 (55.9, 60.1)

Total	5746	99.1	51.3	25.4 (24.2, 26.5)	13.2	39.2 (37.9, 40.4)	52.4 (51.1, 53.7)

Current use of contraception among married women was estimated at 25.4% (24.2, 26.5). Meanwhile, current use of family planning methods was 46.9% (44.3, 49.4), 19.5% (17.9, 21.3) and 16.5% (15.0, 18.1) among urbanites, lowlanders and highlanders, respectively as shown in Table 3. There was no significant difference in the level of contraception between rural lowlanders and highlanders in the district though the difference in unmet need for limiting was not statistically significant.

The unmet need of contraception was estimated to be 52.4% as shown in Table 3. The unmet need for spacing was much higher as compared to limiting. The lowest unmet need for limiting was recorded for urban Butajira while the highest is for highland Butajira. Meanwhile unmet need for spacing was much higher in rural areas compared to urban Butajira. On the other hand, unmet need for spacing was about three-quarter of the total unmet need while the remaining percentage was attributed for limiting. Unmet need for spacing was more than 75% of the total unmet need in lowland and highland Butajira while it contributed to more than two-third of the overall unmet need in urban Butajira. There was statistically significant difference in unmet need for spacing between rural and urban areas.

This study had also revealed that women's desire for children did not significantly decline with increasing size of surviving children. For instance about 70.8% of married women having four surviving children wanted another child. Disaggregation of fertility desire by type of residence showed 62.9%, 73.7% and 71.0% of married women having 4 surviving children in urban, rural lowland and rural highland areas, respectively wanted to have another in the study district (result not shown).

Different reasons for the unmet need of contraception were mentioned by married women of reproductive age group included in this study. Some are reasons for the unmet need while others are socio-cultural values and norms that deter the use of family planning in the community. The majority (43.1%) replied they could not get the method they prefer in the nearby health facility unless they walk a long distance, in some instances up to 18 kilometers. On the other hand, about 22.2% of them expected rejections by religious leaders and their community to practice it. Another 12.6% reported absence of all contraceptives or the ones they preferred in facilities. About 4.8% mentioned lower level service providers' incompetence. Whereas, about 16.8% of married women reported reasons related to the side effect and contraindications of available contraceptive methods in the area such us heart burn, excessive bleeding and their presumption of requirement of balanced diet and optimum work load.

Table 4 showed the bi-variate and multivariate association of various background characteristics of married women with the current use of family planning use in the Butajira district. The odds of current use of family planning was 2.3 (95% CI: 1.66, 3.18) times higher among urbanites compared to highlanders. However, the odds of contraception between highland and lowland Butajira district residents were eliminated when other background characteristics of women were included in the model. There was a positive association between contraception and educational status of women. Women with primary and secondary level of education were about 1.32 (95% CI: 1.12, 1.56) and 1.99 (95% CI: 1.38, 2.88) times respectively more likely to use family planning compared to their uneducated counterparts. Besides, married women who were members of food self deficient households was about 1.58 (95% CI: 1.39, 1.81) times more likely to use family planning compared to their counterparts in food self sufficient households though the association turned statistically not significant when other variables are included.

**Table 4:** The association of background characteristics of married women with current family planning use in Butajira district, 2009 (N = 5746)

Background characteristics		# of married women using FPM	# of married women not using FPM	Crude OR (95%CI)	Adjusted OR (95%CI)
Ecology	Highland	356	1800	1.00	1.00
	Lowland	415	1710	1.23 (1.05, 1.43)*	1.14 (0.96, 1.35)
	Urban	685	777	4.46 (3.82, 5.20)*	2.30 (1.66, 3.18)*

Educational status	Never	800	108	1.00	1.00
	Primary	481	829	2.43 (2.12, 2.78)*	1.32 (1.12, 1.56)*
	Secondary plus	175	3350	6.79 (5.27, 8.73)*	1.99 (1.38, 2.88)*

Food shortage	Yes	387	1563	1.00	1.00
	No	1069	2724	1.58 (1.39, 1.81)*	0.89 (0.76, 1.04)

Dead child	Yes	429	1899	1.00	1.00
	No	966	2117	2.02 (1.77, 2.30)*	1.30 (1.13, 1.50)*

Partner's education	Never	336	1881	1.00	1.00
	Primary	825	2126	2.17 (1.89, 2.50)*	1.32 (1.13, 1.55)*
	Secondary plus	295	280	5.90 (4.83, 7.21)*	1.50 (1.12, 2.01)*

Migration	Non-migrant	823	2756	1.00	1.00
	In-migrant	633	1531	1.38 (1.23, 1.56)*	1.01 (0.87, 1.16)

Livelihood	Farming	794	3499	1.00	1.00
	Trade/service	339	466	3.21 (2.73, 3.76)*	1.13 (0.82, 1.56)
	Civil servant	159	113	6.20 (4.81, 7.99)*	1.25 (0.79, 1.98)
	Other	164	209	3.46 (2.78, 4.30)*	1.15 (0.80, 1.67)

Discussion	Yes	1283	2537	5.23 (4.40, 6.22)*	2.21 (1.80, 2.70)*
	No	167	1728	1.00	1.00

Husband support	yes	1288	2610	4.92 (4.14, 5.85)*	2.59 (2.11, 3.17)*
	No	168	1676	1.0	1.00

*9 5% confidence interval

Meanwhile, the odds of women with no experience of child death were 1.3 (95% CI: 1.13, 1.50) times more likely to use contraceptives compared to those who had dead children. Besides, there was a positive association between partner's educational status of women and contraception. The odds of contraception was 1.32 (95% CI: 1.13, 1.55) and 1.50 (95% CI: 1.12, 2.01) times higher among married women whose partners have primary and secondary plus level of education respectively compared to those who have uneducated partners.

In-migrant women were 1.38 (95% CI: 1.23, 1.56) times more likely to use family planning compared to those whose birth places are the study district though the statistical significance vanished when other factors were included. Furthermore civil servant, handicraft and merchant women were 6.2 (95% CI: 4.81, 7.99), 3.46 (95% CI: 2.78, 4.30) and 3.21 (95% CI: 2.73, 3.76) times respectively more likely to use family planning compared to those whose livelihood were farming although the statistical significant association disappeared when other factors are added in the model.

Discussion about the use of family planning between married women and their partners was significantly associated with contraception in Butajira district. Married women who had discussed about contraception with their partners were 2.2 (95% CI: 1.8, 2.7) times more likely to use the family planning compared to those who did not discussed about family planning. Nevertheless, the odds of contraception was about 2.59 (95% CI: 2.11, 3.17) times higher among married women whose partners support the use of family planning compared to their counterparts.

## Discussion

The 2009 contraceptive prevalence rate (CPR) of 25.4% (95% CI: 24.2, 26.5) among married women in Butajira district is comparable to the regional and national CPR of 25.8 and 28.6, respectively in 2011 [11]. The increase in contraception in recent years could be attributed to the expanding health service coverage and start of an opening of a zonal hospital that has been functional in Butajira district since 2002 which improved the health service delivery [27].

Although contraception increased significantly both nationally and in the study district in recent years it was still much lower than the unachieved target of 60% put for the year 2010 [28]. Moreover, the unmet need of contraception of 52.4% is much higher than 33.8% and 25.3% of the 2005 and 2011 levels, respectively for Ethiopia. Unmet need was estimated at 37.4% and 25.0% for the region in which the study district is located in 2005 and 2011, respectively [10,29]. About 75% of the unmet need recorded in this study is attributed to spacing and the rest is for limiting. The commitment of the Ethiopian government to achieve an 80% contraceptive demand satisfaction by 2015 is far to reach since about 71% of married women who have 4 surviving children desire to have more children and the desire is even higher in rural areas.

Women in the study area mentioned stock out of contraceptives, absence of client preferred methods in facilities, religious pressure, service provider incompetence and side effects of contraceptives such us heart burn, excessive menstrual bleeding and their presumption of requirement of balanced diet and optimum work load as barriers to use family planning methods. Similar reasons of unmet need for contraception were also documented by other studies done elsewhere [30,31]. Inadequacy of methods and counseling were mentioned as reasons for unmet need in a similar study done in Gondar, North Western Ethiopia [32]. The higher unmet need of contraception recorded in this study could be due to the population pressure and poverty driving women in densely populated areas such as the study district and urban centers to look for parity specific controls though service providers cannot cope with the demand [4]. The relatively better level of contraception and lower unmet need among urban married women in Butajira district could be ascribed to accessibility of health services and awareness of the urbanites on family planning methods. Better mass media coverage in urban compared to rural areas might have increased knowledge and practice. Urban women could also be more enlightened and autonomous to utilize health services compared to their counterparts in rural areas [33].

Unlike a study done in Jimma area which showed higher preference of pills, Depo-provera is the most preferred method among current users of family planning methods in the study district [16]. Women in the area could be in favor relatively long acting methods to avoid missing pills or some might have taken long acting methods without the consent of their partners which could be difficult in the case of pills.

Women's educational status is positively associated with contraceptive prevalence rate in the study district. Studies elsewhere revealed a similar pattern of relationship between educational status and maternal health service including family planning utilization [15,34,35]. Longer years of education could probably give married women better chance to understand uses of contraception to reduce fertility, maternal and child morbidity and mortality. It might also increase awareness on the side effects of contraceptive methods to prefer the most convenient ones. Educated women could avoid the negative effects of family planning methods by getting appropriate advice from a service provider thereby increased their consistent use.

Moreover, experience of child death is negatively associated with family planning use in Butajira district. When women have experience(s) of child death they attempt to replace the lost ones and want to have more children [36]. On the other hand, if they perceived low child death in their community they might undergo contraception knowing that most of the children born would reach adulthood. The study district faced periodic droughts associated with epidemics in the context of abject poverty [37] which may be one of the reasons that the level of family planning use is still at a lower level although it is increasing in recent years.

Husband's educational status is associated with family planning use as shown in a study in Eastern Sudan [38]. Men's educational status is directly related to economic and social empowerment which increased exposure to resources such as access to media and utilization of desired health care delivery services. More years of education enabled men to better knowledge of reproductive health behaviors in which case men would be liberal to introduce couple's discussion to use family planning. They would also consider managing family size as something that has to be decided by couples.

Furthermore, this study showed a positive association between couple's discussion on family planning and contraception which was similar to studies elsewhere [20,21]. The role of men in decision making has been instrumental in traditional patrilineal societies like Ethiopia. Men decide almost in every aspect of life including reproductive health service choices. This study revealed significant association between men' support to family planning and its current use among married women. Similar studies done in different parts of Ethiopia showed significant association of men's involvement with family planning methods use [34,35,39].

This study has a drawback in terms of not including men as study participants to explore the difference in perception and practice between men and women and main reasons for this difference. It did not in particular ask the involvement of men in the utilization of family planning and their perspectives towards its use. Moreover, as it is a cross-sectional study it could be difficult to establish cause and effect relationship. It is a study conducted to understand main factors that contribute the current utilization of family planning in a high fertility community with population pressure wherein contraception rate was low which can be mentioned as strength of the study.

In conclusion current family planning use among married women in Butajira district was still low though the unmet need was very high. Barrier to contraception in the area included full stock out and absence of preferred methods, religion, complaints related to providers and methods; and assumption of having proper diet and optimum work load. Factors that were significantly associated with contraception were residential ecology, women and their partners' educational status, experience of child death of women, couple's discussion on contraception and partners' support.

Health systems at various levels in Butajira district and the capacity of their leader in family planning logistics system should be strengthened. The local government should also strive to increase the educational level of residents beyond primary level. The government should also avail family planning methods with appropriate method mix, increase competence of providers, and make awareness on various methods and their temporary side effects. Emphasis should also be given to rural communities.

## Competing interests

The authors declare that they have no competing interests.

## Authors' contributions

WM carried out the research from conception to the write up of the final draft of the article. AW had supervised and critically commented at each stage of the research. All authors read and approved the final manuscript.

## Authors' Information

WM is a PhD candidate in Public Health at School of Public Health in Addis Ababa University

AW is PhD in Biostatistics, advisor of WM and an associate professor at School of Public Health in Addis Ababa University
